# A novel locus (*Bnsdt2*) in a *TFL1* homologue sustaining determinate growth in *Brassica napus*

**DOI:** 10.1186/s12870-021-03348-0

**Published:** 2021-12-03

**Authors:** Kaixiang Li, Liang Xu, Yongpeng Jia, Cuiping Chen, Yanmei Yao, Haidong Liu, Dezhi Du

**Affiliations:** Academy of Agricultural and Forestry Sciences of Qinghai University, Key Laboratory of Spring Rape Genetic Improvement of Qinghai Province, Rapeseed Research and Development Center of Qinghai Province, Xining, 810016 Qinghai China

**Keywords:** *Brassica napus*, Determinate inflorescence, Agronomic traits, *Bnsdt2*, qRT-PCR

## Abstract

**Background:**

The determinate growth habits is beneficial for plant architecture modification and the development of crops cultivars suited to mechanized production systems. Which play an important role in the genetic improvement of crops. In *Brassica napus*, a determinate inflorescence strain (4769) has been discovered among doubled haploid (DH) lines obtained from a spring *B. napus* × winter *B. napus* cross, but there are few reports on it. We fine mapped a determinate inflorescence locus, and evaluated the effect of the determinate growth habit on agronomic traits.

**Results:**

In this study, we assessed the effect of the determinate growth habit on agronomic traits. The results showed that determinacy is beneficial for reducing plant height and flowering time, advancing maturity, enhancing lodging resistance, increasing plant branches and maintaining productivity. Genetic analysis in the determinate (4769) and indeterminate (2982) genotypes revealed that two independently inherited recessive genes (*Bnsdt1, Bnsdt2*) are responsible for this determinate growth trait. *Bnsdt2* was subsequently mapped in BC_2_ and BC_3_ populations derived from the combination 2982 × 4769. *Bnsdt2* could be delimited to an approximately 122.9 kb region between 68,586.2 kb and 68,709.1 kb on C09. BLAST analysis of these candidate intervals showed that *chrC09g006434* (*BnaC09.TFL1*) is homologous to *TFL1* of *A. thaliana*. Sequence analysis of two alleles identified two non-synonymous SNPs (T136C, G141C) in the first exon of *BnaC09.TFL1*, resulting in two amino acid substitutions (Phe46Leu, Leu47Phe). Subsequently, qRT-PCR revealed that *BnaC09.TFL1* expression in shoot apexes was significantly higher in NIL-4769 than in 4769, suggesting its essential role in sustaining the indeterminate growth habit.

**Conclusions:**

In this study, the novel locus *Bnsdt2*, a recessive genes for determinate inflorescence in *B. napus*, was fine-mapped to a 68,586.2 kb - 68,709.1 kb interval on C09. The annotated genes *chrC09g006434* (*BnaC09.TFL1*) that may be responsible for inflorescence traits were found.

**Supplementary Information:**

The online version contains supplementary material available at 10.1186/s12870-021-03348-0.

## Background

In higher plants, inflorescences can be divided into indeterminate inflorescences and determinate inflorescences. To date, it has been found inflorescences with determinate growth habits have an important effect on agronomic traits in many plants. The determinate inflorescence mutant *tfl1* was screened from wild-type *Arabidopsis thaliana* using ethylmethane sulphonate (EMS) treatment. The *tfl1* mutant shoot apex produced a terminal flower, preventing further shoot apical meristem (SAM) differentiation, and exhibited reduced plant height, early flowering and a clear large-seed phenotype [1–3]. In leguminous crops, plants with determinate inflorescence, controlled by *Vrdet1* (*TFL1* homologue), showed earlier ripening of pods than those with indeterminate inflorescence [4]. In sesame, studies of the determinate inflorescence mutant *DS899* showed that compared with plants with indeterminate inflorescence, this plant showed reduced plant height (by approximately 26.4%), increased 1000-grain weight, and shortened growth and flowering periods, which played an important role in the yield improvement of sesame [5, 6]. Kaur and Banga assessed the phenotypic variation in 125 newly identified determinate *B. juncea* strains. The results showed that among the 125 determinate *B. juncea* strains, many determinate strains were superior to the indeterminate commercial varieties in some traits (earliness, plant height, pod number, yield, and oil content) [7]. In general, determinate inflorescences have the characteristics of early flowering, reduced plant height and advanced maturity, which play an important role in the genetic improvement of crops.

The *TFL1* gene and its homologue play an important role in determinate inflorescence. The *TFL1* gene affects stem apex expansion and structure through interaction with the *LFY*, *AP1* and *FT* genes, which are expressed in the apical meristem of *A. thaliana*. *TFL1* can maintain the attributes of the inflorescence meristem (IM), while *LFY* and *AP1* act as recognition genes in the floral meristem. In the IM, *TFL1* mRNA is restricted to the inner cells, and the *TFL1* protein is a mobile signal that is uniformly distributed throughout the meristem. In the floral meristem, the signal feedback of *LFY* and *AP1* stimulates the movement of the *TFL1* protein and maintains normal expression of the genetic attributes of the floral meristem, thus forming an indeterminate inflorescence that can continuously produce flowers [8]. In addition, the durations of the early vegetative and reproductive stages of the *TFL1* mutant were greatly shortened due to the overexpression of *FT* [9]. Although *FT* and *TFL1* have highly similar amino acid residues, these genes have opposite functions [10–13]. In cucumber, *CsTFL1* interacts with *CsNOT2a* to inhibit determinate inflorescences and terminal flower formation, but low expression of *CsTFL1* causes non-interaction with *CsNOT2a* to produce determinate growth [14]. In *Phaseolus vulgaris*, *PvTFL1y* restored normal indeterminate growth, similar to that of wild-type *A. thaliana*, when it was introduced into the *tfl1* mutant, which has determinate inflorescences [15]. Therefore, the *tfl1* gene is essential for the maintenance and control of determinate plant growth.

Rapeseed is one of the most important oil crops in China, among which *B. napus* is the most important cultivated species. Du et al. [16] found a determinate inflorescence mutant in microspore culture of *B. napus*. A genetic analysis showed that the determinate trait is controlled by one recessive gene, *Bnsdt1*. The *Bnsdt1* gene was located on chromosome A10 of *B. napus*, and *BnA10.tfl1* was identified as the gene controlling the trait of determinate inflorescence [16, 17]. However, we found that this trait is not controlled by one recessive gene in breeding. Therefore, a new study on the determinate inflorescence mutant 4769 was carried out. In the present study, the determinate inflorescence mutant 4769 of *B. napus* was analysed. We found a novel locus (*Bnsdt2*) of determinate inflorescence and performed fine mapping and cloning. The results will contribute to a better understanding of determinacy.

## Results

### Effects of determinate growth habit on agronomic traits

**Fig. 1:**
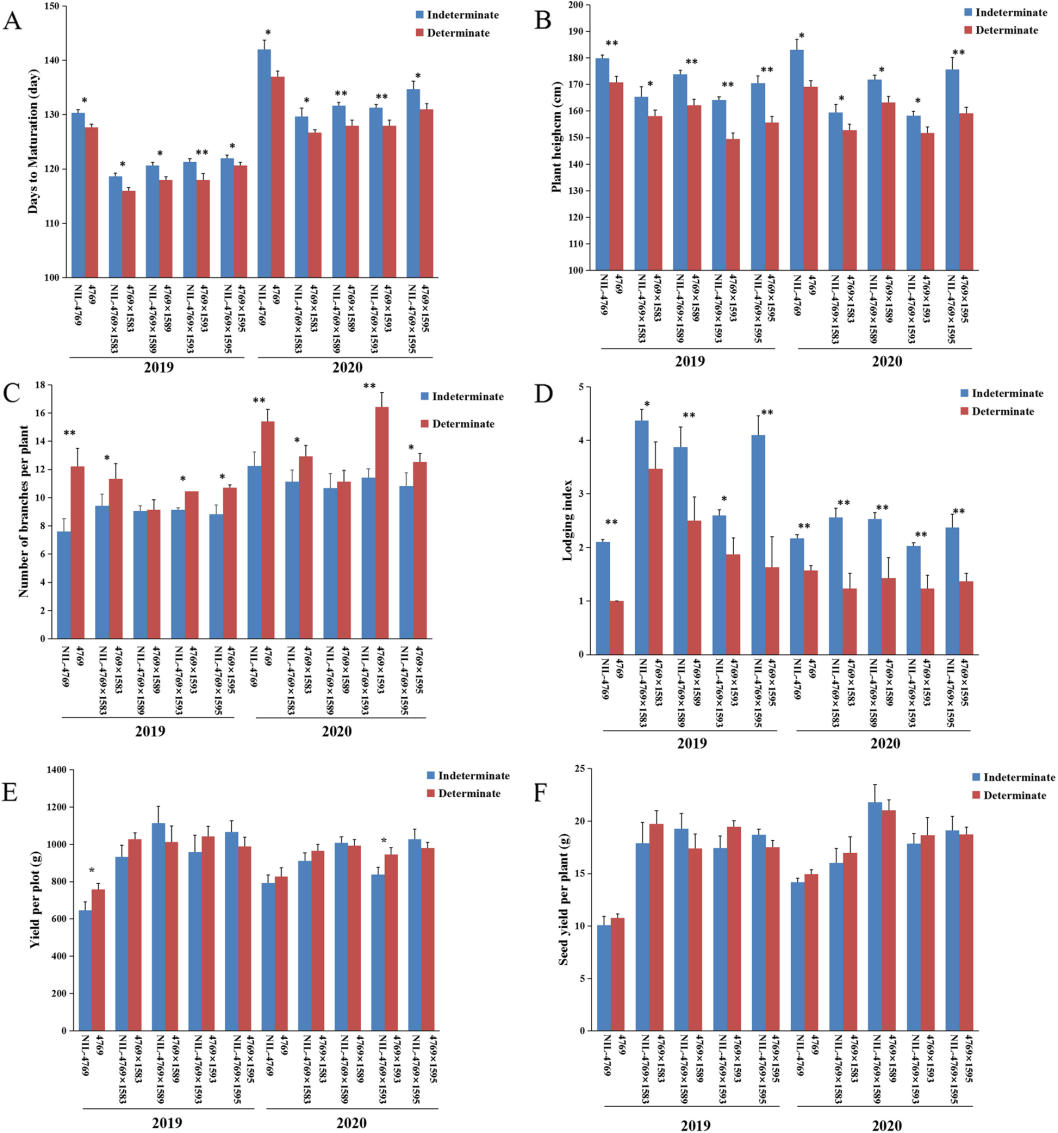
Phenotypes of determinate and indeterminate agronomic traits in the near-isogenic lines (4769 and NIL-4769) and eight hybrid combinations over two years (2019 and 2020). **A** Days to maturation, **B** plant height, **C** number of branches per plant, **D** lodging index, **E** yield per plot, **F** seed yield per plant

To understand the effect of determinate growth habit on agronomic traits, we investigated some agronomic traits of the near-isogenic lines of determinate/indeterminate (4769 and NIL-4769) and eight hybrid combinations over 2 years (2019 and 2020) under standard agricultural conditions in Xining, China. First, these traits (initial flowering, final flowering and maturation) were surveyed and analysed for each plot. For the near-isogenic lines of the determinate/indeterminate plants (4769 and NIL-4769) and eight hybrid combinations, the number of days to initial flowering was not different between determinate and indeterminate lines in the 2 years. However, the final flowering and maturation times were significantly different between the determinate and indeterminate varieties in the 2 years (the determinate preceded the indeterminate varieties by 2–5 days) (Fig. 1A, Table S1). These results indicated that the determinate growth habit could shorten the time to the final flowering period and the mature period. Table S1 presents information about the three traits (initial flowering period, final flowering period, and mature period).

Subsequently, some yield-related traits were surveyed and analysed. The results showed that the determinate inflorescences had no effect on five yield-related traits (number of siliques per plant, seeds per silique, thousand-seed weight, seed yield per plant, yield per plot) in the near-isogenic lines of determinate/indeterminate plants (4769 and NIL-4769) and eight hybrid combinations during 2019 and 2020 (Fig. 1E, F; Table S2). Indeed, these traits did not significantly differ between the determinate and indeterminate groups. However, three other yield-related traits (plant height, number of branches per plant and lodging resistance) were significantly different between the determinate and indeterminate plants (Fig. 1B, C, D; Table S2).

The analysis above showed that compared with the indeterminate plant, determinate *B. napus* exhibited advancement of final flowering and maturity, reduced plant height, enhanced lodging resistance and increased number of branches per plant. However, it exhibited no penalty effect on the siliques per plant, thousand-seed weight, seeds per silique, seed yield per plant or yield per plot.

### Genetic analysis

The segregation pattern of the growth habit using F_2_ and BC_1_ populations was investigated and analysed. Between the determinate and indeterminate genotypes, all F_1_ individuals displayed a complete indeterminate phenotype, indicating the dominance of the indeterminate growth habit over the determinate growth habit. In 2982 × 4769 F_2_ plants, 499 and 30 plants had indeterminate and determinate growth, respectively, which was consistent with the 15:1 segregation ratio. The BC_1_ plants were segregated in an approximate 3:1 ratio (BC_1_: indeterminate:determinate = 213:62) (Table 1). The results indicated that the determinate growth habit was controlled by two independently inherited recessive genes. Subsequently, we scanned the BC_1_ plants of the combination 2982 × 4769 with some markers closely linked to the *Bnsdt1* gene, and the results showed that it contained the *BnSDT1*/*Bnsdt1* locus. Therefore, we hypothesize that there was a new recessive locus, and the new determinate inflorescence gene was tentatively designated the *Bnsdt2* gene.

**Table 1:** Segregation of inflorescence traits in the BC_1_ and F_2_ progenies

Combination	Population	No. of INDT. plants	No. of DT. plants	Expected ratio	χ2 value
2982 × 4769	F_2_	499	30	15:1	0.30
	BC_1_F_1_	213	62	3:1	0.88

Χ^2^_0.05,1_ = 3.84

### Primary mapping of the *Bnsdt2* gene

**Fig. 2:**
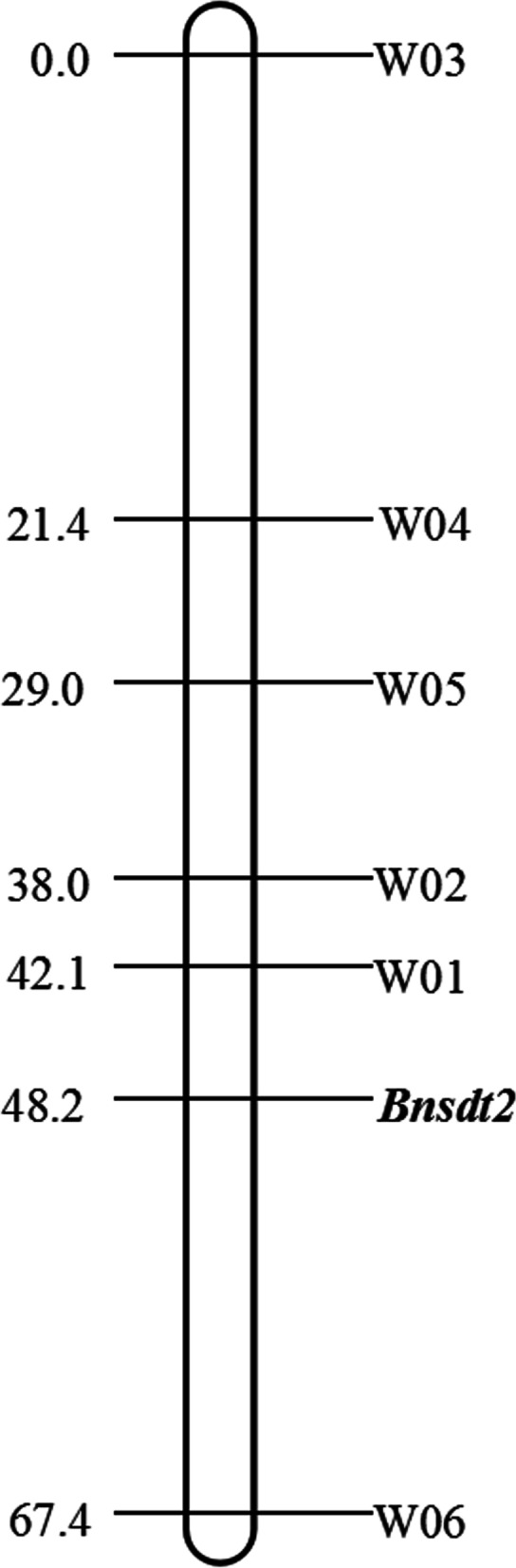
Genetic linkage map of the *Bnsdt2* gene

Five hundred - seventeen individuals of BC_2_ population were established by the ((2982 × 4769) × 4769) × 4769 cross to detect molecular markers linked to the *Bnsdt2* gene. Six polymorphic markers linked to the *Bnsdt2* gene were identified from 512 P + 3/M + 3 (256 P01–16/MC01–16 + 256 P01–16/MG01–16) and 512 EA + 3/M + 3 (256 EA01–16/MC01–16 + 256 EA01–16/MG01–16) AFLP primer combinations and named W01-W06 (Table 2). Next, a genetic linkage map was constructed and the results showed that W01-W05 and W06 were located on both sides of the *Bnsdt2* gene (Fig. 2). Among these flanking markers of the *Bnsdt2* gene, W01 and W06 were the most closely linked and were 6.1 and 19.2 cM away from the *Bnsdt2* gene, respectively. Subsequently, six specific AFLP fragments were successfully cloned and sequenced. By BLAST analysis against the BRAD database (http://www.brassicadb.org/brad/), five AFLP markers (W02-W06) presented sequence homology to chrCnn_random of *B. napus* (the Darmor-bzh reference genome (Bna41: *Brassica_napus* V4.1)) (Table 3). The W01 was located on C06 of *B. napus*. Therefore, it can be inferred that the *Bnsdt2* gene is located in the C genome of *B. napus* (chrCnn_random was not assembled on the C chromosome), presenting great difficulty in mapping.

**Table 2:** Description of AFLP markers tightly linked to the *Bnsdt2* gene

AFLP marker	Primer combination	Size of marker (bp)	Map distance (cM)
W01	P-AGA/M-GCG	136	6.1
W02	P-ACT/M-GAA	205	10.2
W03	E-AAT/M-CGT	268	48.2
W04	E-ATT/M-CGT	99	26.8
W05	E-AGT/M-CAG	323	19.2
W06	E-ACC/M-CTA	184	19.2

P *PstI* primer, 5′-GACTGCGTACATGCAG-3′; E *EcoRI* primer, 5′-GACTGCGTACCAATT C-3′; M *MseI* primer, 5′-GATGAGTCCTGAGTAA-3′

**Table 3:** Results of BLASTN searches using sequences from the AFLP fragments

AFLP marker	Linkage group (position)	Identity	Homologous gene in *B. napus*
W01	chrC06 (33277106–33,277,022)	153, 8E-36, 83/85 (97%)	
W02	chrCnn_random (49341751–49,341,629)	228, 2e-15, 83/85 (97%)	GSBRNA2T00052245001
W03	chrCnn_random (2405842–2,406,040)	389, e-106, 209/212 (98%)	GSBRNA2T00038164001
W04	chrCnn_random (70990276–70,990,314)	78, 3e-13, 39/39 (100%)	
W05	chrCnn_random (11845806–11,846,104)	593, e-168, 299/299 (100%)	
W06	chrCnn_random (60484242–60,484,083)	317,3 e-85, 160/160 (100%)	

In the “Linkage group” column, the numbers in the brackets indicate the position of the *B. napus* homologous sequence corresponding to our sequence. In the “Identity” column, the first and second numbers are the score and the expected value (E value) given by BLASTN, respectively; the fraction indicates the numbers of residues that are identical in our sequence and the corresponding *B. napus* sequence

### Fine mapping of the *Bnsdt2* gene

According to the physical locations of these markers on chrCnn_random, the *Bnsdt2* gene is likely located in the 2.0 Mb–12.0 Mb and 49.0 Mb–71.0 Mb regions of chrCnn_random of *B. napus* (Table 3). Subsequently, we downloaded the region sequence from BRAD and developed 98 SSR markers. Finally, three SSR markers were found and named BnW07 to BnW09 (Table 4).

**Table 4:** Information about the markers that were closely linked to *Bnsdt2*

Type of marker	Name	Size of marker (bp)	Physical position on chrCnn_random of Darmor-bzh (kb)	Physical position on C09 of *B. oleracea* (kb)	Physical position on C09 of *B. napus* NY7 (kb)
SSR	BnW07	292	9405.6	39,378.9	68,437.4
SSR	BnW08	214	9400.5	39,373.8	68,431.4
SSR	BnW09	237	9480.5	39,458.2	68,518.8
SSR	BoW10	183	9344.5	39,458.2	68,375.4
SSR	BoW11	184	9537.1	39,483.8	68,586.2
SSR	BoW12	181	9543.9	39,490.5	68,593.0
SSR	BnW13	193	2245.1	39,592.0	68,740.4
SSR	BnW14	195	2233.1	39,580.0	68,728.2
SSR	BnW15	195	2233.1	39,580.0	68,728.2
SSR	BnW16	231	2222.8	39,570.0	68,717.8
SSR	BnW17	239	9538.5	39,485.0	68,587.4
SSR	BnW18	292	9480.7	39,458.4	68,519.1
SSR	BnW19	214	9538.5	39,485.2	68,587.7
SSR	BnW20	237	9545.8	39,491.6	68,594.9
SSR	BnW21	183	71,187.6	39,561.9	68,709.1
SSR	BnW22	471	2216.9	39,564.1	68,712.1
SSR	BnW23	212	2217.1	39,564.3	68,712.2

*B. napus* is an allotetraploid species (AACC) formed by *Brassica campestris* (AA) and *Brassica oleracea* (CC), and the *Bnsdt2* gene is locked on the C chromosome. Therefore, we sequenced the specific fragments of these three SSR markers (BnW07 to BnW09) and compared them with the *B. oleracea* genome (CC). We found that these three markers were located on C09 (39 M–40 M) of the *B. oleracea* genome (BOL11: *Brassica oleracea* V1.1) (Table 4). In this interval, we developed 65 SSR markers and three SSR markers (BoW10 to BoW12) had polymorphism. Therefore, we hypothesize that the *Bnsdt2* gene was located on C09. Genomic sequence information for Ningyou 7 (NY7) (http://ibi.zju.edu.cn/bnpedigome/) was obtained by third-generation genome sequencing technology in 2019. We subjected these six SSR markers to BLAST analysis, and the results showed that the *Bnsdt2* gene is located on C09 of *B. napus* and delimited to the region around 68 Mb. Subsequently, five polymorphic SSR markers (from 71 SSR loci) were developed in this interval (BnW13-BnW17). Therefore, the *Bnsdt2* gene was finally mapped to C09 of *B. napus*.

**Fig. 3:**
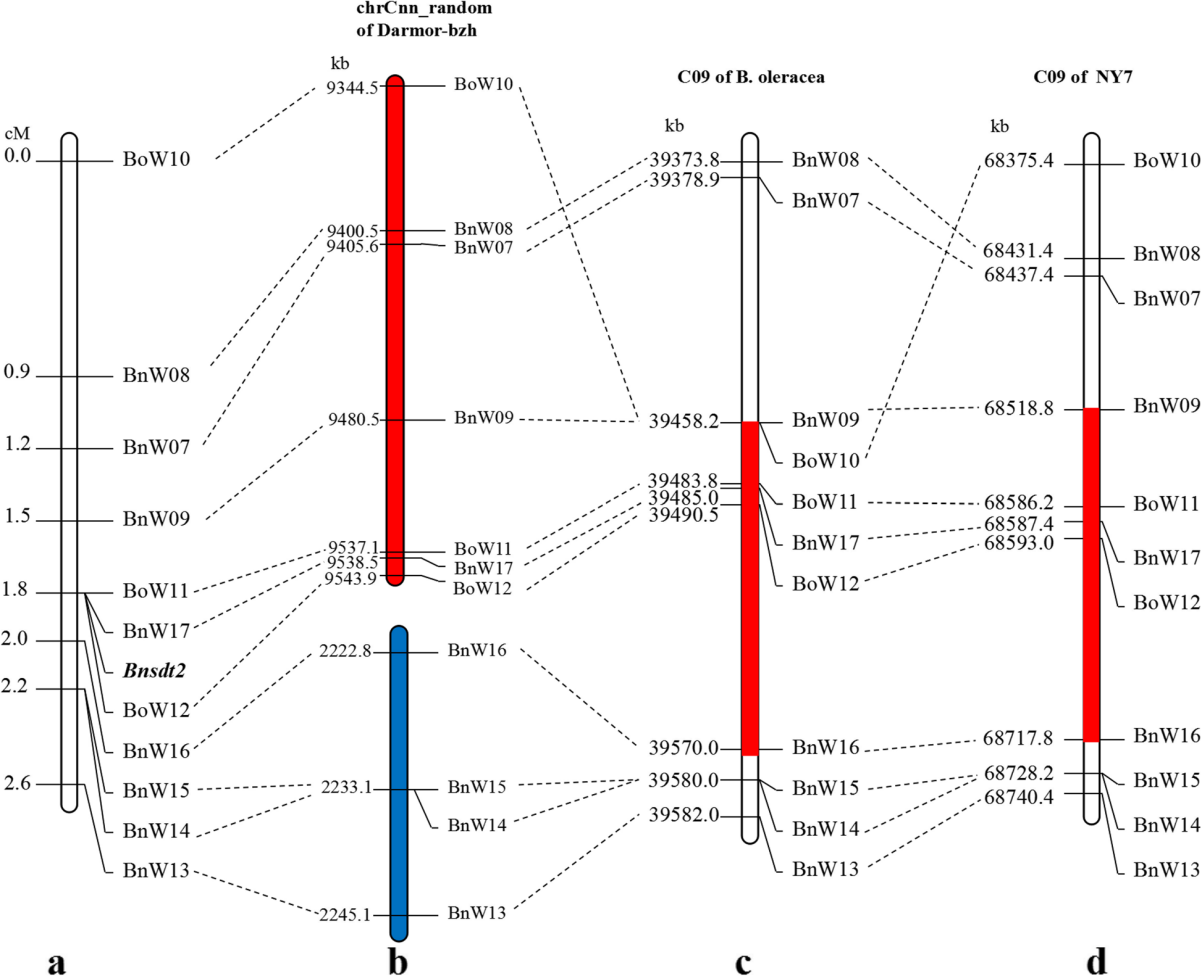
**a** Partial linkage map of the region surrounding the *Bnsdt2* gene. **b** Partial physical map of chrCnn_random of Darmor-bzh showing the homologues of the mapped marker sequences. **c** Partial physical map of C09 of *B. oleracea,*the red region indicates candidate intervals. **d** Partial physical map of C09 of NY7*,* the red region indicates candidate intervals

All individuals of the BC_2_ population were identified using these polymorphic markers on C09 of *B. napus*. The results showed that four markers (BnW07-BnW09 and BoW10) were located on one side of the *Bnsdt2* gene, four markers (BnW13-BnW16) were located on the other side of the *Bnsdt2* gene and three markers (BoW11, BoW12 and BnW17) co-segregated with the *Bnsdt2* gene (Fig. 3). Among the markers flanking the *Bnsdt2* gene, BnW09 and BnW16 were the most closely linked and were 0.3 cM and 0.2 cM from the *Bnsdt2* gene, respectively (Table 4). The closely linked markers mentioned above were used for BLAST analysis against BRAD (http://www.brassicadb.org/brad/). The results showed that all of the markers were located on chrCnn_random of the *Darmor-bzh* reference genome (Bna41: *Brassica_napus* V4.1) (Table 4). More interestingly, four markers were located near 2 Mb, and another seven markers were located near 9 Mb and turned upside down (Fig. 3). Then, these markers were used for BLAST analysis against the reference genome of *B. oleracea* (BOL11: *Brassica oleracea* V1.1), and the results showed that these markers were located on C09 of *B. oleracea* (near 39 Mb). All the markers corresponding to these markers on our map appeared in the same order as those on C09 of *B. napus* (Fig. 3). Based on this order, the genomic region containing the *Bnsdt2* gene was delimited to an interval of approximately 199 kb between 68,518.8 kb and 68,717.8 kb on C09.

**Fig. 4:**
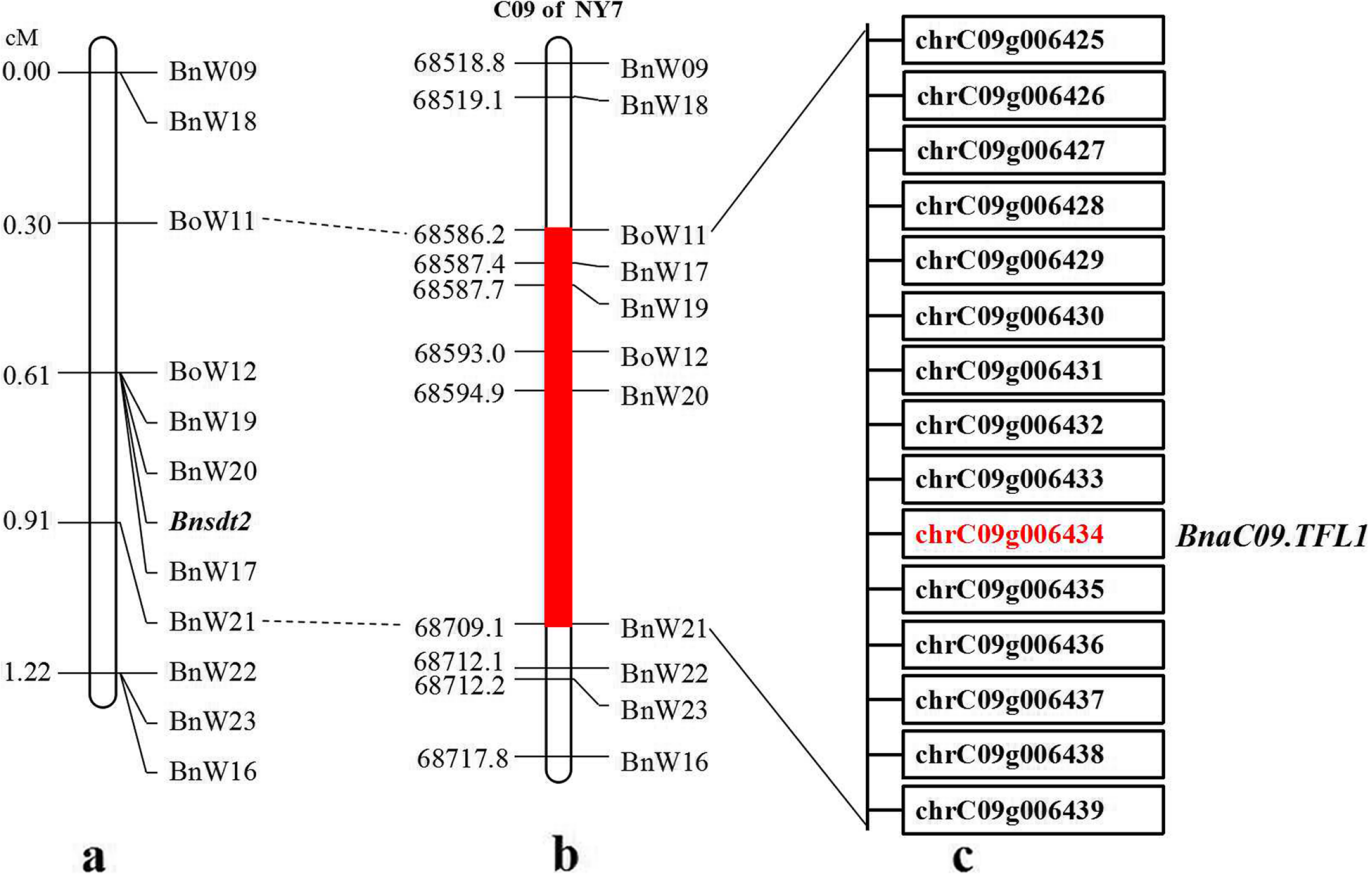
Analysis of candidate intervals for the *Bnsdt2* gene on chromosome C09 of *B. napus*. **a** A partial genetic linkage map of the region around the *Bnsdt2* gene. **b** A partial physical map of linkage markers around the *Bnsdt2* gene. The red region indicates candidate intervals. **c** Results of a BLAST analysis using sequences from candidate intervals against the NY7 genome

To narrow the target region of *Bnsdt2*, we constructed 1426 individuals of BC_3_ population. Subsequently, six SSR markers (BnW18-BnW23) were developed and identified from the interval of 68,518.8 kb - 68,717.8 kb on C09 (Table 4). These six SSR markers and some previously used markers (BnW09, BoW11-BoW12, BnW16-BnW17) were employed to screen 1426 BC_3_ individuals. The results showed that the *Bnsdt2* gene was positioned between BoW11 and BnW21. By BLAST analysis against BnPedigome, the *Bnsdt2* gene was delimited to an interval of approximately 122.9 kb between 68,586.2 kb and 68,709.1 kb on C09 of *B. napus* (Fig. 4). In addition, we also found two co-dominant markers (BnW09 and BnW18) from the above markers, which could be used in molecular marker-assisted breeding of rapeseed.

### Dissection of the *Bnsdt2* target region

The candidate sequences were submitted to BnPedigome (http://ibi.zju.edu.cn/bnpedigome/) and TAIR database (http://www.arabidopsis.org/) for BLAST analysis. The results showed that the candidate region included 15 predicted genes and were homologous with 14 *Arabidopsis* genes (Table 5). According to the TAIR database of gene annotation of these genes, the *chrC09g006434* (*BnaC09.TFL1*) gene is homologous to the *AT5G03840* gene (Table 5). The *AT5G03840* is the *TERMINAL FLOWER 1* (*TFL1*) gene of *A. thaliana*, which encodes a phosphatidylethanolamine binding protein (PEBP). In *Arabidopsis TFL1* mutants, the determinate inflorescence can be formed at the top of the inflorescence. Therefore, we inferred that *chrC09g006434* (*BnaC09.TFL1*) gene was the most important candidate gene of the *Bnsdt2*, and select this gene for further study.

**Table 5:** Results of BLASTN searches using the candidate interval gene

Gene of *B. napus*	Homologous gene in *A. thaliana*	Putative function
chrC09g006425	AT5G03940	Chloroplast signal recognition particle 54 kDa subunit
chrC09g006426	AT5G03905	Iron-sulphur cluster biosynthesis family protein
chrC09g006427	AT5G03900	Unknown
chrC09g006428	AT5G03890	Unknown
chrC09g006429	AT5G03880	Thioredoxin family protein
chrC09g006430	AT4G12370	Unknown
chrC09g006431	AT5G03850	Nucleic acid binding, OB-fold-like protein
chrC09g006432	AT4G30340	Diacylglycerol kinase 7
chrC09g006433	AT5G03850	Nucleic acid binding, OB-fold-like protein
chrC09g006434	AT5G03840	PEBP (phosphatidylethanolamine-binding protein) family protein (*TFL1*)
chrC09g006435	AT2G29900	Presenilin-2
chrC09g006436	AT5G40820	Ataxia telangiectasia-mutated and RAD3-related
chrC09g006437	AT5G03795	Exostosin family protein
chrC09g006438	AT5G03790	Homeobox 51
chrC09g006439	AT1G32200	Phospholipid/glycerol acyltransferase family protein

### Sequence analysis of *BnaC09.TFL1*

**Fig. 5:**
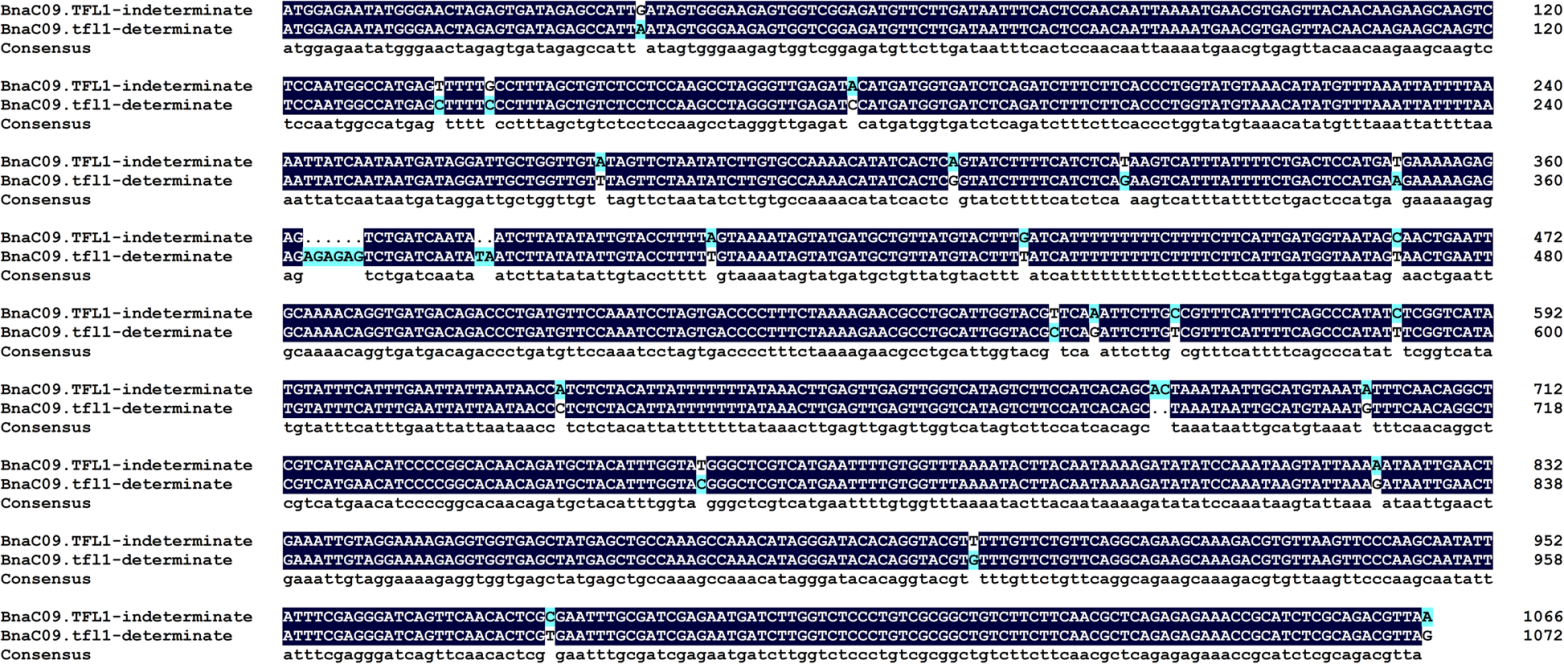
gDNA sequence alignment between indeterminate and determinate sequences

**Fig. 6:**
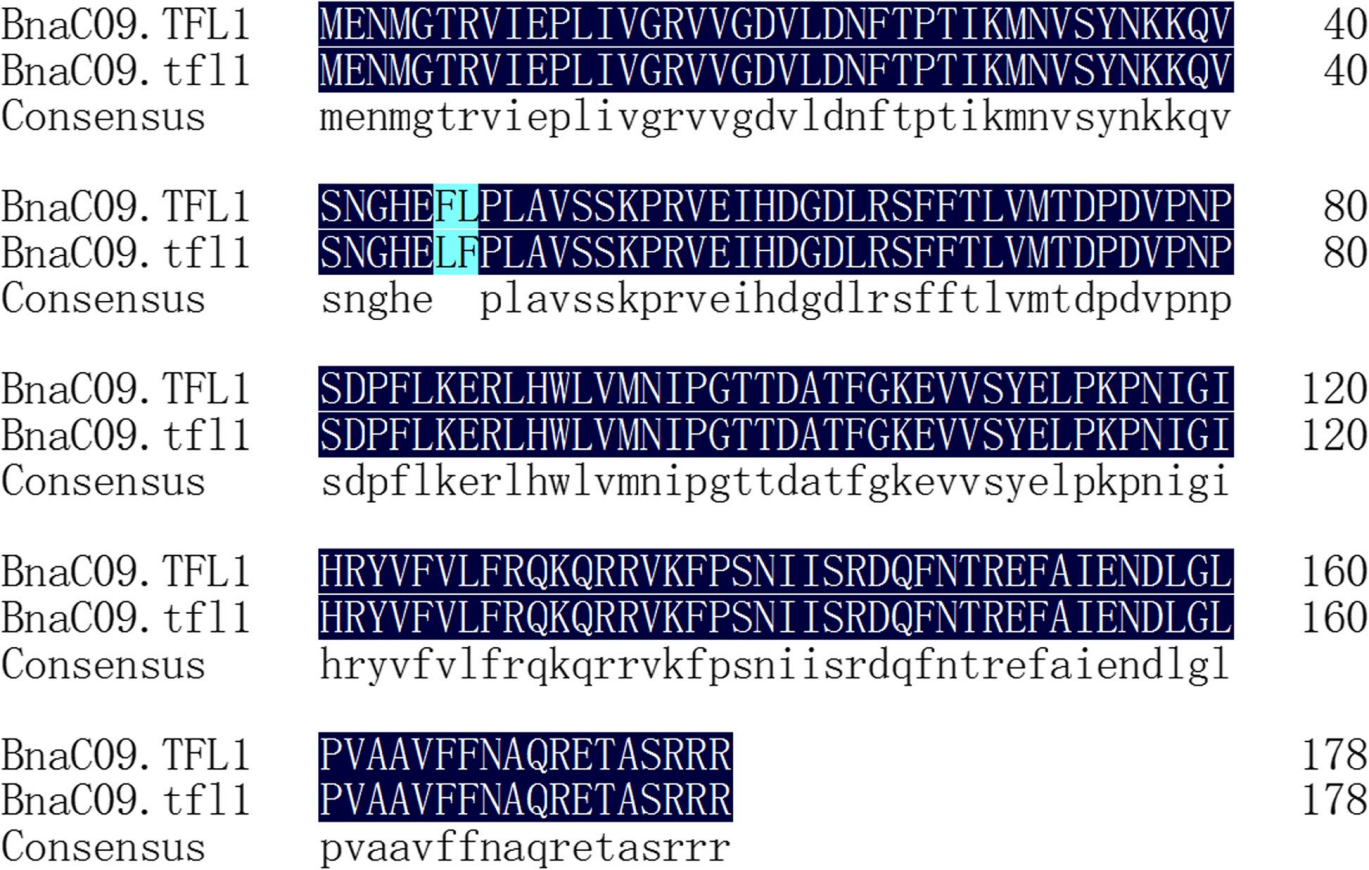
Amino acid sequence alignment between indeterminate and determinate sequences

The sequences of gDNA and CDS of the *BnaC09.TFL1* gene were successfully obtained from parents and near-isogenic lines. Sequence alignment analysis showed that there were no sequence differences between the parents and the corresponding near-isogenic lines with the same inflorescence traits. Subsequently, the gDNA sequences of determinate and indeterminate inflorescences were analyzed and the results showed that there were many differences between gDNA of determinate and indeterminate inflorescences. It contains 22 single-base mutations,1 insertion (2 bases) and 2 deletions (6 bases and 2 bases) (Fig. 5). Amino acid sequence prediction and analysis of the *BnaC09.TFL1* gene showed that two non-synonymous SNP mutations (T136C, G141C) were found in the first exon of the *BnaC09.TFL1* gene. It leads to two amino acid substitutions (Phe replaces Leu and Leu replaces Phe) (Fig. 6), while the other amino acids were conserved (98.88%).

### Expression pattern analysis of *BnaC09.TFL1*

**Fig. 7:**
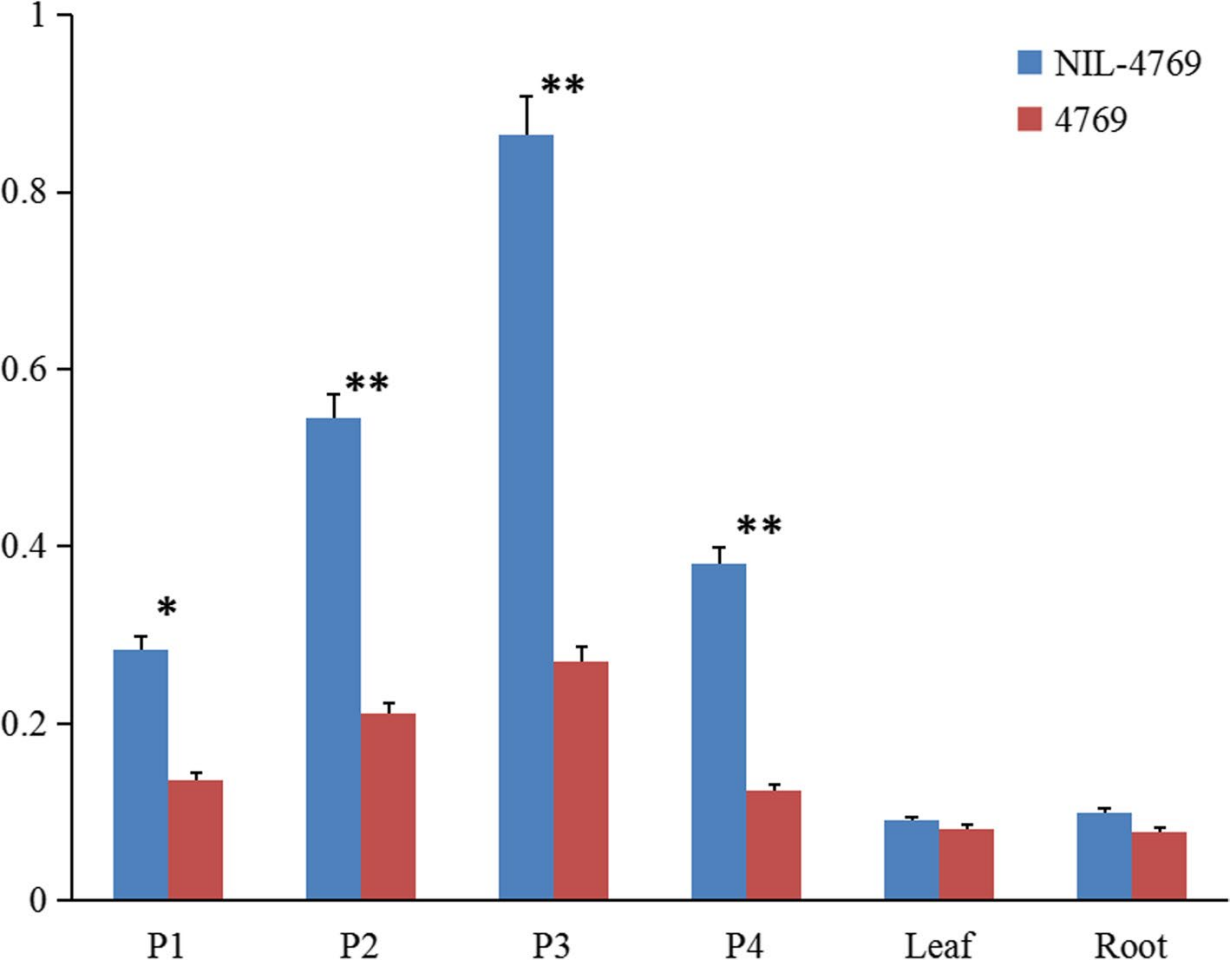
Relative expression of *BnaC09.TFL1* in different organs of the near-isogenic lines of determinate/indeterminate inflorescence (4769 and NIL-4769) plants. P1: 2-leaf seedlings, P2: 4-leaf seedlings, P3: budding period, P4: initial flowering. The sampling time of different organs (leaf, root) was P1. **, highly significant difference between NIL-4769 and 4769 plants; *, significant difference between NIL-4769 and 4769 plants

We studied the expression pattern of *BnaC09.TFL1* in near-isogenic lines (4769 and NIL-4769) using qRT-PCR. In order to avoid the effect of homologous copies, the qRT-PCR primers was designed at specific locations of the CDS sequences of determinate and indeterminate (Table S3). The results of expression analysis showed that the *BnaC09.TFL1* expression had little difference in leaf and root organs, whereas the difference in the shoot apex was notable. The total expression level of *BnaC09.TFL1* in the shoot apex was significantly higher than that in other organs, indicating that *BnaC09.TFL1* was specifically expressed in the shoot apex. In addition, the expression of *BnaC09.TFL1* in NIL-4769 was significantly higher than that in 4769, especially in P2 and P3, which represent the early stage before the formation of inflorescence traits (Fig. 7). Therefore, it is reasonable to hypothesize that *BnaC09.TFL1* is a potential candidate gene for inflorescence traits.

## Discussion

Our study showed that the determinacy have no effect on initial flowering, but could advance final flowering and maturation (2–5 days) and shorten the growth period in both near-isogenic lines and hybrid combinations. At present, the phenomenon of shorten growth period had also been found in the determinacy of other plants. Such as *A. thaliana* [18, 19], *Antirrhinum majus* [20], *Solanum lycopersicum* [10] and *Sesamum indicum* [5, 6]. But the determinacy of these plants shorten the growth period by flowering early, while the determinacy of *B.napus* did not bloom early. The reason of short growth period of the determinacy in *B.napus* was analyzed. It was believed that the determinacy broke the advantage of inflorescence apex, accelerated the growth and development of each inflorescence, increased the number of flowering per unit time, shortened the flowering time of the plant. Compared with the indeterminacy, the determinacy could terminate flowering earlier and shorten the whole growth period. In addition, the determinacy of *B.napus* could reduce plant height and enhance lodging resistance. This was consistent with the research results in *Sesamum indicum* [6], *Glycine max Merr* [21], *Vicia faba* [22], *B. juncea* [7]. The determinacy of *B.napus* could enhance the cause of the lodging resistance were analyzed. We hold the opinion that due to the degradation of the top of the determinacy, the length of each inflorescence became shorter, the plant height decreased, and each inflorescence was basically at the same level height, so that the whole plant was in a balanced state without tilting to a certain direction. Then, we studied the effect of the determinacy on yield, and the results showed that the determinacy had no negative effect on yield. This was consistent with the research results of other plants with determinate growth habit. We analyzed the causes of its formation and found that the determinacy could increase number of branches per plant, but had no effect on the siliques per plant, thousand-seed weight, seeds per silique, seed yield per plant or yield per plot. This indicated that the determinacy have the same number of siliques per plant as indeterminacy by increasing the number of branches, when the number of silique per branch becomes smaller. And it could ensure that the yield does not decrease.

With the discovery of determinate inflorescence mutants in *A. thaliana* [18], *Antirrhinum majus* [20], *Sesamum indicum* [5], *Vigna radiata* [23], *Glycine max* [24], *Cucumis sativus* [25] and *B. juncea* [7], among others, the genetics of determinate inflorescences have been investigated in these plants, and determinacy is controlled by one recessive gene. The determinate inflorescence strain (4769) of *B. napus* has been discovered among DH lines obtained from a spring *B. napus* × winter *B. napus* cross. Genetic analysis revealed that the determinate inflorescence resulting from the two combinations of 2014 × 4769 and 2092 × 4769 was controlled by one recessive gene, *Bnsdt1*. However, we found that it is not controlled by one recessive gene in breeding. Therefore, the DH lines 2982 and 4769 of *B. napus* were used as materials in the present study. The results showed that the determinate inflorescence (4769) resulting from the combination of 2982 × 4769 cells was controlled by two independently inherited recessive genes, namely, *Bnsdt1* and *Bnsdt2*. The *Bnsdt2* gene is a newly recessive locus. Zhang et al. [26] studied a natural mutant strain *FM8* of *B. napus* with a determinate inflorescence, and genetic analysis showed that the inheritance of the determinate inflorescence was controlled by the interaction of two recessive genes and one recessive epistasis suppressor gene. The above findings indicate that the genetic pattern of determinate inflorescence mutants is different in *B. napus*. This may be caused by the following two reasons: on the one hand, the mutant type of determinate inflorescence is different; on the other hand, the parent materials of indeterminate inflorescence are closely related to determinate inflorescence when the genetic pattern of determinate inflorescence traits is analysed, which results in loss of the genetic locus.

In previous studies, most of the rapeseed genes that control quality traits were isolated by map-based cloning approaches [7, 27, 28]. We used this method to map the determinate trait of *B. napus*. In the early stages of the experiment, we could preliminarily inferred that the *Bnsdt2* gene was located in the C genome of *B. napus*. But we didn’t know exactly where the *Bnsdt2* gene was located on which chromosome of the C genome. Then we took the genomic sequence information of *B. oleracea* as reference and mapped the *Bnsdt2* gene to the C09 chromosome. With the released of Ningyou 7 (NY7) genome sequence information in 2019. Finally, the *Bnsdt2* gene was delimited to an interval of approximately 122.9 kb between 68,586.2 kb and 68,709.1 kb on C09 of *B. napus*. The above findings indicated that a suitable reference genome for gene mapping is very important. In the case of allopolyploid plants, the reference genome of the original ancestors could be referred to in the absence of a suitable reference genome. In addition, according to gene annotation of these genes in the intervals, we found that the gene *chrC09g006434* (*BnaC09.TFL1*) was highly similar to the *TFL1* gene in *A. thaliana*. Sequence analysis of *BnaC09.TFL1* showed that the gDNA sequences had many differences between indeterminate and determinate sequences. The sequence contains 2 deletions (of 6 bases and 2 bases), 1 insert (of 2 bases) and 22 single base mutations (Fig. 7) and identifies two non-synonymous SNP mutations (T136C, G141C) in the first exon of *BnaC09.TFL1*, resulting in two amino acid substitutions (Phe46Leu, Leu47Phe). Subsequently, we performed an analysis of the expression pattern of *BnaC09.TFL1*. The results showed that *BnaC09.TFL1* was specifically expressed in the shoot apex. Therefore, it is reasonable to postulate that *BnaC09.TFL1* is a potential candidate gene for the inflorescence trait.

In summary, these studies are important because we are not familiar with determinate inflorescences in *B. napus*. They also provide a basis for future breeding and gene cloning.

## Conclusions

In this study, we identified a novel locus *Bnsdt2*, a recessive genes for determinate inflorescence in *B. napus*, was fine-mapped to a 68,586.2 kb - 68,709.1 kb interval on C09. The annotated genes *chrC09g006434* (*BnaC09.TFL1*) was identified as suitable candidate genes for *Bnsdt2* according to sequence analysis and qRT-PCR analysis. In addition, the determinate growth habit has positive effects on agronomic traits. It provides a new insight for plant architecture modification and the development of rapeseed cultivars suited to mechanized production systems.

## Methods

### Plant material and population construction

**Fig. 8:**
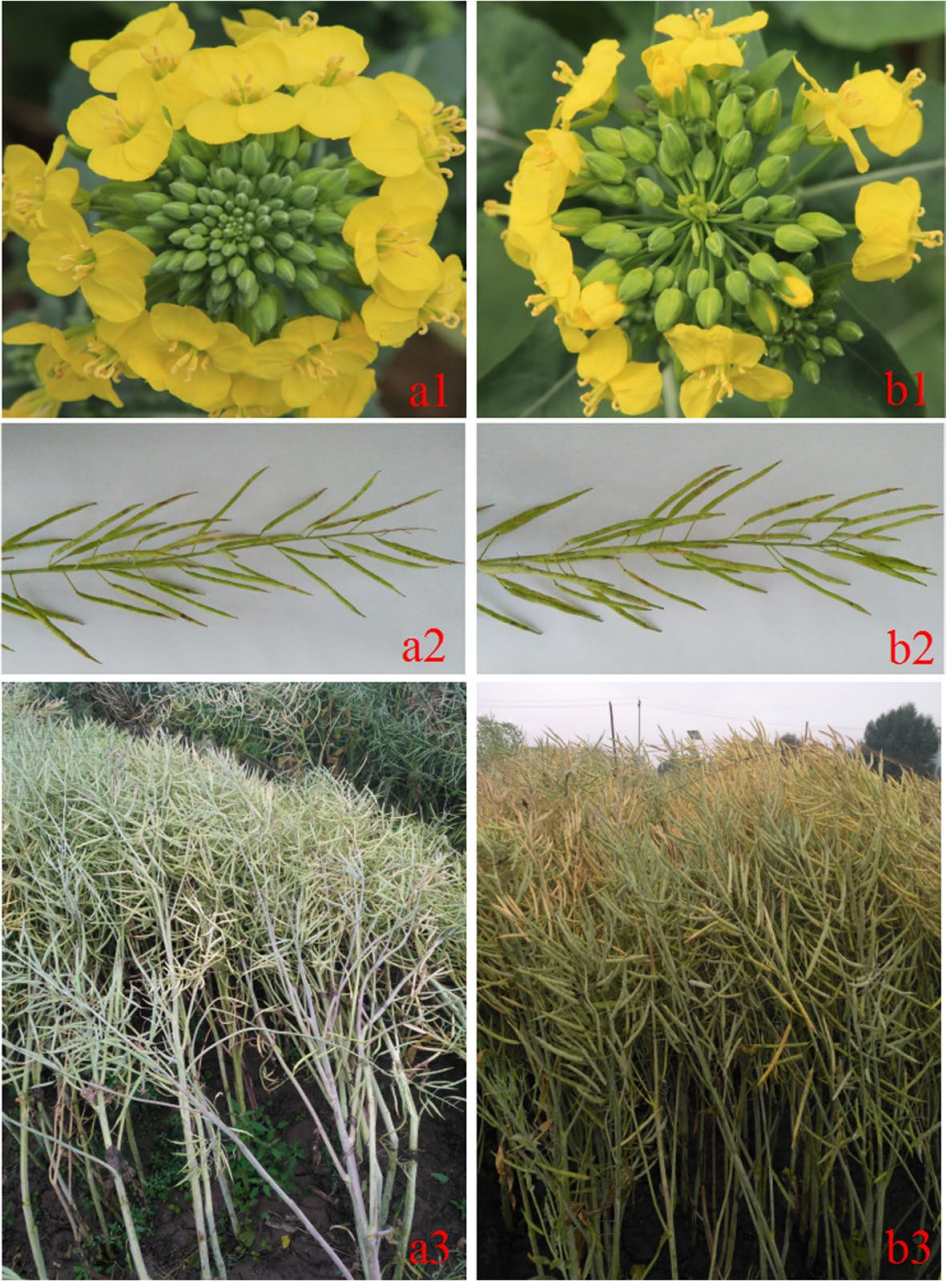
Two IM development phenotypes in near-isogenic lines (4769 and NIL-4769). The images show the stem apexes of two genotypes at flowering and maturity. **a1** Shows the indeterminate genotype. **b1** Shows the determinate genotype, with variation in fruit clusters at the shoot apex. **a2**, **a3** Shows the indeterminate genotype at maturity, and **b2**, **b3** presents the determinate genotype at maturity

The DH lines 2982 and 4769 of *B. napus* were used as materials in the present study. The inflorescence of 2982 is indeterminate, and the inflorescence of 4769 is determinate (from the progeny of microspore culture [spring *B. napus* × winter *B. napus*] F_1_). Inheritance of the inflorescence trait was studied with F_2_ and BC_1_ populations. The inflorescence traits were investigated during the flowering period. Figure 8 shows two IM development phenotypes in near-isogenic lines.

The *Bnsdt2* locus was separated by using the BC_1_ population derived from a 2982 × 4769 cross. In the BC_1_ population, we selected 10 indeterminate individuals (These 10 indeterminate individual were screened by some markers closely linked to *Bnsdt1.*Theoretically, the genotype of these 10 indeterminate individual were *Bnsdt1Bnsdt1BnSDT2Bnsdt2.*) and backcrosses with 4769 individuals to establish a BC_2_ isolate line (Fig. S1), which was sown in the Xining Experimental Base (Qinhai Province, China). The inflorescence traits of each line were investigated. Due to the existence of recombinant plants and the number plants too little in some isolated lines. The results showed that the separation ratio of indeterminate to determinate was 1:1 in 3 out of 10 lines. Therefore, we hypothesized that these three strains contain a dominant locus (*BnSDT1* or *BnSDT2*). To verify that the three strains contained the *BnSDT1* or *BnSDT2* locus, 72 indeterminate individuals (each strain containing 24 individuals) were selected for scanning by the co-dominant markers closely linked to the *Bnsdt1* gene. The results showed that the co-dominant markers did not show different bands in 72 indeterminate plants and showed the same banding pattern as the determinate. This indicates that the three strains are controlled by another locus, so their genotypes are speculated to be aaBb (*Bnsdt1Bnsdt1BnSDT2Bnsdt2*) (Fig. S1). Therefore, three strains containing the *BnSDT2* locus were isolated from the BC_2_ population and used for mapping the *Bnsdt2* gene. To conduct fine mapping of the *Bnsdt2* gene, in the three BC_2_ isolate strains, we selected 15 indeterminate individuals (five indeterminate individuals each strains) and backcrosses with 4769 individuals to establish 1426 individuals of the BC_3_ population.. In order to assess the agronomic performance of the determinacy, we selected five indeterminate individuals (The background recovery rate was 100% by scanning of 38 SSR markers) self-cross twice in the BC_3_ population, and constructed near-isogenic lines 4769 (determinate) and NIL-4769 (indeterminate).

### Field trial and trait evaluation

Eight hybrid combinations were operated between the near-isogenic lines 4769 (determinate)/NIL-4769 (indeterminate) and determinate lines 1583, 1589, 1593, and 1595. The near-isogenic lines and eight hybrid combinations were grown in Xining (Qinhai Province, China) for 2 years (2019 and 2020). A randomized complete block design was conducted with three replications. Each plot was planted with five rows, with 12 plants in each row and a distance of 15 cm between plants within each row and 30 cm between rows. During the entire reproductive stage, the initial flowering, final flowering and maturation were recorded for each plot. At the mature stage, 8–10 open-pollinated plants from each plot were selected to test agronomic traits, and the seeds were threshed by hand. For agronomic traits, the main tests included plant height (PH), number of primary branches (NPB), number of secondary branches (NSB), number of branches per plant (NBP), number of siliques per plant (NSP), seeds per silique (SS), thousand seed weight (TSW), seed yield per plant (SYP) and yield per plot (YP). Lodging was investigated before maturation of the plant, and the lodging index was calculated following the methods described by Shi [29]. The data of these traits were analysed by using SPSS statistical software [30].

### DNA extraction and amplified fragment length polymorphism (AFLP) analysis

DNA was extracted individually from fresh leaves using the cetyltrimethylammonium bromide (CTAB) method [31]. Two determinate and indeterminate bulks were constructed using equivalent amounts of DNA from 12 determinate and 12 indeterminate individuals in BC_2_ populations, respectively. Subsequently, two determinate and indeterminate bulks were used for bulked segregant analysis (BSA) [32] in combination with the AFLP technique. Genomic DNA was cleaved with the restriction enzymes *PstI, EcorI* and *MseI*. Pre-amplification was performed using the AFLP primers P0/MC, P0/MG, EA/MC, and EA/MG. The preamplified product was diluted (1:30) and used for selective amplification. AFLP amplification was performed as described by Vos et al. [33]. The product of selective amplification was separated and silver stained as described for AFLP markers.

### AFLP and SSR marker sequencing and identification of *B. napus* synteny

The expected AFLP and SSR bands were collected and purified following the methods described by Yi et al. [34]. The purified products were ligated into the pMD18-T Easy vector (TaKaRa). Subsequently, M13-specific primers were used to detect the transformed clones. For each fragment, three positive clones were randomly selected and sequenced by Shanghai Sangon Biotechnology Corporation (Shanghai, China). After genetic mapping of The BC_2_ and BC_3_ populations, the sequences of these markers (six AFLP and 17 SSR markers) were used to identify putative homologous sequences within the *B. napus* genome. BLAST analysis was performed using the Brassica Database (BRAD) (http://www.brassicadb.org/brad/) and BnPedigome Database (http://ibi.zju.edu.cn/bnpedigome/).

### Development of SSR markers

Because the chromosome sequence of *B. napus* has been published, the DNA sequence of the segment was downloaded from BRAD and the BnPedigome database according to the marker-locked region. SSR loci were detected using SSRHunter 1.3 software [35], and the SSR primers were designed using Primer 3 software [36]. SSR amplification was performed as described by Lowe et al. [37]. The amplified products were separated on a 6% denaturing polyacrylamide gel.

### Mapping

The BC_2_ populations (547 individuals) and BC_3_ populations (1426 individuals) were used for mapping the *Bnsdt2* gene. The AFLP data, SSR markers and individual phenotypes were analysed with the JoinMap 4/MapDraw program [38, 39], and a partial linkage map of the region on the chromosome spanning the *Bnsdt2* gene was constructed. The genetic distance (in cM) was calculated using the Kosambi function.

### Cloning and expression analysis of the candidate gene

The *BnaC09.TFL1* gene was amplified from the genomic DNA of 2982 and 4769 using the primers described in Table S3. The gDNA sequences were analysed using DNAMAN 8.0, and amino acid sequence prediction and analysis of the *BnaC09.TFL1* gene were performed using Premier 5. For real-time qRT-PCR analysis, the shoot apex of near-isogenic lines (4769 and NIL-4769) were sampled at different developmental stages (P1: 2-leaf seedlings, P2: 4-leaf seedlings, P3: budding period, P4: initial flowering), frozen quickly in liquid nitrogen and stored at − 80 °C for RNA extraction. Total RNA was isolated by the TaKaRa MiniBEST Plant RNA Extraction Kit (TaKaRa, Dalian, China). RNA was also extracted from the roots and leaves of 2-leaf seedlings. The first-strand cDNA was synthesized using the PrimeScript™ RT Reagent Kit (TaKaRa, Dalian, China) according to the manufacturer’s protocol. To measure the mRNA levels of genes, qRT-PCR was conducted using a LightCycler® 480 Instrument II (Roche, Basel, Switzerland) with SYBR Green Mix (TaKaRa, Dalian, China). The actin gene was selected as the reference gene for relative quantification of the candidate gene. The PCR conditions were as follows: 95 °C for 2 min followed by 45 cycles of 95 °C for 10 s and 60 °C for 30 s. A melting curve analysis was also performed to confirm the specificity of the primers. The data were processed using the 2^−ΔΔCT^ method [40].

### Statement

In this study, The methods were carried out in accordance to the current laws of China and international guidelines and legislation. The plant materials (or cultivars) were collected and preserved, with the appropriate permissions, by the Academy of Agricultural and Forestry Sciences of Qinghai University in China. All necessary permissions for planting and investigating these plant materials (or cultivars) were obtained from Academy of Agricultural and Forestry Sciences of Qinghai University in China, and the collection and research of these plant materials (or cultivars) have complied with the Convention on the Trade in Endangered Species of Wild Fauna and Flora.

## Supplementary Information

**Additional file 1: Fig. S1.** Population construction. **Table S1.** Flowering analysis of near-isogenic lines ear-isogenic lines (NIL-4769 and 4769) and eight hybrid combinations. **Table S2.** Analysis of agronomic traits of near-isogenic lines (NIL-4769 and 4769) and eight hybrid combinations. **Table S3.** The primer sequences of gDNA, cDNA and qRT-PCR.

## Data Availability

All data used during the study are included in this published article and its additional files.

## Abbreviations

DH: Doubled haploid
qRT-PCR: Quantitative real-time PCR
AFLP: Amplified fragment length polymorphism
SSR: Simple sequence repeat
