# Supplementary material for: A novel locus (Bnsdt2) in a TFL1 homologue sustaining determinate growth in Brassica napus
Source: BMC Plant Biol. 2021 Dec 3;21:568. doi: 10.1186/s12870-021-03348-0 (PMC8641158; doi:10.1186/s12870-021-03348-0)
Supplement: Supplementary file 1 — Additional file 1: Fig. S1. Population construction. Table S1. Flowering analysis of near-isogenic lines ear-isogenic lines (NIL-4769 and 4769) and eight hybrid combinations. Table S2. Analysis of agronomic traits of near-isogenic lines (NIL-4769 and 4769) and eight hybrid combinations. Table S3. The primer sequences of gDNA, cDNA and qRT-PCR. [file 12870_2021_3348_MOESM1_ESM.pdf]

A novel locus (Bnsdt2) in a TFL1 homologue sustaining determinate growth in Brassica napus

Kaixiang Li†, Liang Xu†, Yongpeng Jia, Cuiping Chen, Yanmei Yao, Haiding Liu, Dezhi Du*

(Academy of Agricultural and Forestry Sciences of Qinghai University, Key Laboratory of Spring Rape Genetic Improvement of Qinghai Province, Rapeseed Research and Development Center of Qinghai Province, Xining 810016, Qinghai, China)

* Correspondence: qhurape@126.com

† Kaixiang Li and Liang Xu have contributed equally to this article.

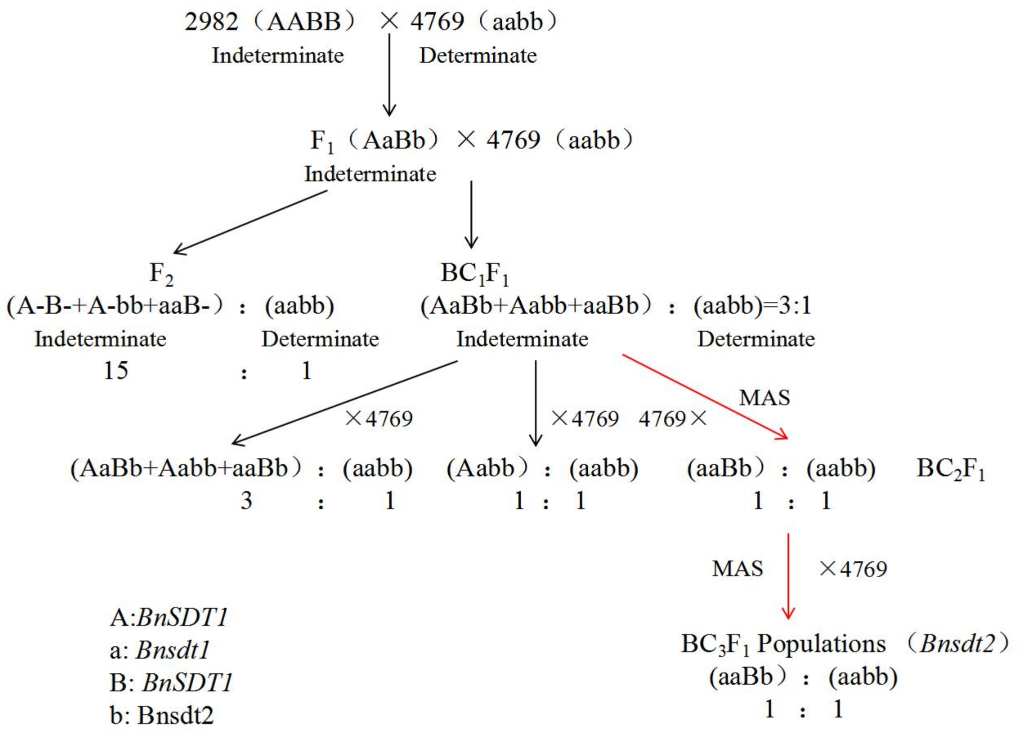

Fig. S1. population construction

Table S1 Flowering analysis of near-isogenic lines ear-isogenic lines (NIL-4769 and4769) and eight hybrid combinations

year	NIL and hybrid combination	Days to flowering	Days to end flowering	Days to Maturation
2019	NIL-4769	73.33±0.58a	105.67±0.58a	130.33±0.58a
	4769	71.67±0.58a	103.00±1.00b	127.67±0.58b
	NIL-4769×1583	62.67±0.00a	94.00±1.00A	118.67±0.58a
	4769×1583	63.67±0.58a	90.33±0.58B	116.00±0.58b
	NIL-4769×1589	64.00±0.58a	94.67±0.58A	120.67±0.58a
	4769×1589	64.33±0.58a	91.67±1.00B	118.00±0.58b
	NIL-4769×1593	63.67±0.58a	93.67±1.00a	121.33±0.58A
	4769×1593	63.00±0.58a	90.33±0.58b	118.00±1.15B
	NIL-4769×1595	64.67±0.00a	94.00±0.58a	122.00±0.58a
	4769×1595	63.33±0.58a	92.00±0.58b	120.67±0.58b
2020	NIL-4769	79.33±0.58a	112.33±1.15a	142.00±1.73a
	4769	80.00±1.53a	107.00±1.00b	137.00±1.00b
	NIL-4769×1583	69.67±1.00a	100.00±1.00a	129.67±1.53a
	4769×1583	70.67±1.53a	97.33±0.58b	126.67±0.58b
	NIL-4769×1589	70.00±0.58a	99.67±0.58A	131.67±0.58A
	4769×1589	70.33±1.73a	97.67±0.58B	128.00±1.00B
	NIL-4769×1593	70.67±1.53a	99.67±1.53a	131.33±0.58A
	4769×1593	70.00±0.58a	96.33±0.58b	128.00±1.00B
	NIL-4769×1595	70.67±0.58a	101.00±1.00a	134.67±1.53a
	4769×1595	70.33±1.00a	98.67±0.58b	131.00±1.00b

Table S2　Analysis of agronomic traits of near-isogenic lines (NIL-4769 and4769) and eight hybrid combinations

Year	NIL and hybrid combination	PH(cm)	NPB	NSB	NBP	NSP	SNS	TSW(g)	SYP(g)	YP(g)	Lodging index
2019	NIL-4769	179.81±2.45A	5.17±0.35b	2.44±0.90B	7.60±0.90B	214.57±8.05a	21.54±1.87a	3.80±0.14a	10.09±0.83a	645.56±45.66b	2.10±0.05A
	4769	170.78±1.33B	6.10±0.01a	6.11±1.29A	12.22±1.29A	250.50±21.31a	17.88±0.84b	3.63±0.19a	10.79±0.35a	757.18±33.24a	1.00±0.00B
	NIL-4769×1583	165.43±2.37a	4.44±0.07a	4.99±0.46B	9.43±0.81b	261.37±13.87a	23.01±1.15a	4.01±0.03A	17.89±2.00a	932.27±61.90a	4.37±0.21a
	4769×1583	158.11±3.68b	5.00±0.44a	6.33±0.37A	11.33±1.08a	289.33±17.47a	24.91±0.34a	3.70±0.06B	19.73±1.28a	1026.29±36.06a	3.47±0.60b
	NIL-4769×1589	173.80±2.84A	4.89±0.28a	4.18±0.61a	9.06±0.36a	287.06±4.51a	22.73±0.60a	4.03±0.13a	19.27±1.47a	1113.82±90.24a	3.87±0.38A
	4769×1589	162.23±1.58B	4.52±0.49a	4.63±0.24a	9.15±0.71a	274.52±11.27a	22.61±0.82a	3.91±0.05a	17.38±1.38a	1012.43±84.81a	2.50±0.44B
	NIL-4769×1593	164.09±3.21A	4.73±0.20a	4.09±0.20B	9.15±0.13b	319.14±9.53a	17.97±0.21a	4.09±0.13a	17.45±1.14a	959.18±89.84a	2.60±0.10a
	4769×1593	149.52±1.34B	5.00±0.18a	5.46±0.39A	10.46±0.34a	327.70±16.07a	17.22±0.73a	4.16±0.12a	19.47±0.57a	1041.37±54.74a	1.87±0.31b
	NIL-4769×1595	170.47±2.08A	5.25±0.48a	4.36±0.17B	8.82±0.65B	352.44±7.03a	18.46±1.30a	4.01±0.12a	18.70±0.52a	1066.07±59.58a	4.10±0.36A
	4769×1595	155.76±2.73B	4.96±0.21a	5.76±0.10A	10.71±0.20A	343.69±15.11a	17.34±0.64a	3.86±0.13a	17.53±0.62a	989.16±47.90a	1.63±0.57B
2020	NIL-4769	183.08±6.44a	7.17±0.35a	5.07±0.73B	12.24±1.00b	299.77±25.90a	22.56±0.84a	3.77±0.04a	14.17±0.40a	793.22±42.93a	2.17±0.07A
	4769	169.17±3.86b	7.63±0.48a	7.78±0.50A	15.40±0.86a	327.94±29.07a	24.00±1.19a	3.71±0.07a	14.96±0.39a	826.10±47.50a	1.57±0.09B
	NIL-4769×1583	159.47±3.11a	5.40±0.30A	5.72±0.79a	11.13±0.83b	234.90±25.28a	22.23±0.44a	3.94±0.12a	16.01±1.40a	909.90±44.30a	2.56±0.17A
	4769×1583	152.84±2.96b	6.55±0.24B	6.37±0.80a	12.92±0.78a	260.37±19.14a	22.49±1.68a	3.80±0.08a	16.97±1.55a	963.77±35.40a	1.23±0.29B
	NIL-4769×1589	171.88±4.45a	5.79±0.81a	4.89±0.21B	10.69±1.00a	260.49±36.40a	23.85±0.67a	4.03±0.13a	21.79±1.71a	1008.73±31.70a	2.53±0.12A
	4769×1589	163.32±1.72b	5.83±0.64a	5.97±0.31A	11.13±0.81a	238.40±15.94a	23.56±0.89a	3.98±0.10a	21.04±0.99a	992.10±32.88a	1.43±0.38B
	NIL-4769×1593	158.28±2.57a	6.03±0.50B	5.38±0.20B	11.41±0.64B	301.90±16.32a	17.13±0.96a	4.32±0.18a	17.85±0.98a	837.51±38.04b	2.03±0.06A
	4769×1593	151.72±1.63b	8.06±0.41A	8.36±0.87A	16.42±1.03A	311.12±20.28a	16.65±0.73a	4.40±0.15a	18.67±1.69a	944.52±37.66a	1.23±0.25B
	NIL-4769×1595	175.66±4.55A	6.36±0.50a	4.47±0.45b	10.83±0.92b	207.03±18.66a	24.81±1.32a	4.06±0.09a	19.12±1.35a	1027.03±53.46a	2.37±0.25A
	4769×1595	159.14±4.50B	6.83±0.32a	5.68±0.39a	12.52±0.60a	222.58±13.48a	21.28±1.15b	3.90±0.15a	18.74±0.67a	980.48±29.48a	1.37±0.15B

Table S3 The primer sequences used in this study

Primer	F	R	Function
BnaC09.TFL1-ORF	TTGTCCGAATCTTACAACCA	TTTGATGGACACAGACGTAA	Cloning the gDNA of *BnaC09.TFL1* and *BnaC09.tfl1*
BnaC09.TFL1-CDS	ATGGAGAATATGGGAACTAGAGTGA	T/CTAACGTCTGCGAGATGCGG	Cloning the cDNA of *BnaC09.TFL1* and *BnaC09.tfl1*
qBnaC09.TFL1	CCCCGGCACAACAGATGCTACA	TGAGCGTTGAAGAAGACAGCCG	quantitative real-time PCR
Actin	TGGACGGTGCTGCTTCTGAGTGTATG	GCTGCATCCGACGGCTGAGGTA	quantitative real-time PCR; the reference gene
